# Hepatitis E Virus (HEV) Infection among Humans and Animals: Epidemiology, Clinical Characteristics, Treatment, and Prevention

**DOI:** 10.3390/pathogens12070931

**Published:** 2023-07-12

**Authors:** Jelena Prpić, Magdalena Baymakova

**Affiliations:** 1 Croatian Veterinary Institute, Savska Cesta 143, 10000 Zagreb, Croatia; 2 Department of Infectious Diseases, Military Medical Academy, 1606 Sofia, Bulgaria

The public health significance of hepatitis E is very important. According to the World Health Organization (WHO), there are an estimated 20 million hepatitis E virus (HEV) infections worldwide every year, leading to an estimated 3.3 million symptomatic cases of hepatitis E [1]. The WHO estimates that HEV infection caused approximately 44,000 deaths in 2015, which represents 3.3% of mortality rates due to viral hepatitis [1]. The HEV was identified in 1983 by a Soviet (Russian) scientific group with team leader Mikhail S. Balayan [2]. For years, the HEV was believed to be endemic in places with poor biosecurity and hygiene measures, and was therefore considered to be travel-related. Nowadays, the HEV represents an emerging zoonotic infection in many European countries [1]. It is estimated that 5–15% of all acute viral hepatitis infections of unknown origin in Europe are caused by the HEV [1].

The HEV is a single-stranded positive-sense RNA virus [3]. According to the last classification released in 2022 on the International Committee on Taxonomy of Viruses (ICTV), the HEV is classified in the *Hepeviridae* family (divided in two subfamilies: *Orthohepevirinae* and *Parahepevirinae*) [3,4]. The *Orthohepevirinae* subfamily includes four genera: *Avihepevirus* genus (member species: *Avihepevirus egretti* and *Avihepevirus magniiecur*), *Chirohepevirus* genus (member species: *Chirohepevirus desmodi*, *Chirohepevirus eptesici*, and *Chirohepevirus rhinolophi*), *Paslahepevirus* genus (member species: *Paslahepevirus alci* and *Paslahepevirus balayani*), and *Rocahepevirus* genus (member species: *Rocahepevirus eothenomi* and *Rocahepevirus ratti*) [3]. The *Parahepevirinae* subfamily includes only one genus—*Piscihepevirus heenan* species (cut-throat trout virus) [5]. *Avihepevirus magniiecur* species (avian HEV) was detected only in birds and its strains have been divided into four genotypes: genotype 1 (gt 1) is restricted to Australia; gt 2 and gt 3 have been detected in Asia, Europe, and the USA; and gt 4 has been found in Asia and Hungary [6,7,8,9,10,11,12]. The *Chirohepevirus* genus (bat HEV) was found only in bats with no scientific evidence of transmission to humans [13]. The *Paslahepevirus* genus has a different host range (humans, domestic, and wild mammals) [14]. *Paslahepevirus balayani* species have been assigned to eight genotypes: HEV gt 1 (humans; Southern Asia), HEV gt 2 (humans; Africa and Mexico), HEV gt 3 and HEV gt 4 (bottlenose dolphins, cattle, deer, goats, humans, pigs, rabbits, rats, sheep, etc.; America, Asia, and Europe), HEV gt 5 and HEV gt 6 (wild boars; Japan), HEV gt 7 (*Camelus dromedarius*, human; United Arab Emirates), and HEV gt 8 (*Camelus bactrianus*; China) [14,15,16,17,18,19,20,21,22,23,24,25]. The *Rocahepevirus* genus (rat HEV/ferret HEV/vole HEV) was found in rodents, shrews, and carnivores [3]. *Rocahepevirus ratti* species (gt C1) were detected in eulipotyphlids (musk shrew, *Suncus murinus*), humans, and rodents (*Bandicota indica*, *Rattus* sp.) [26,27,28,29,30,31,32,33]. *Rocahepevirus ratti* species (gt C2) were found among mustelids (ferret, mink, etc.) [34,35].

The HEV often presents undetectable viral pathology in infected organisms (among humans and/or animals). Usually, the viral load remains low, and viral shedding is shorter (acute HEV infection) or longer (chronic HEV infection) [36]. In immunocompetent persons under the age of 50 years, acute HEV infection is asymptomatic and takes a mild clinical form or moderate clinical form in comparison with individuals over 50 years of age; the severe clinical form is more often observed in them [36]. Chronic HEV infection is rare and is most commonly reported in immunosuppressed and immunocompromised persons (patients with solid organ transplantation; hematologic malignancy patients; HIV-positive individuals; inflammatory bowel disease patients; persons with rheumatic diseases; etc.) [36]. Numerous monitoring studies have been performed in Europe in the past in order to determine HEV circulation in the animal population. Domestic pigs and wild boars are considered the main reservoirs of the virus and a potential source of zoonotic transmissions based on serological and molecular results [37,38,39,40,41,42]. HEV infection is mainly transmitted through the consumption of contaminated food or water [43]. Direct contact transmission has been demonstrated in domestic pigs [44,45]. There are reports available that associate HEV infection with the consumption of raw or undercooked food products of pigs, wild boar, deer, or contaminated shellfish [46,47,48,49]. In comparison with the general population, a statistically higher seroprevalence is found in pig farmeers and veterinarians [50]. This suggests that contact exposure to domestic pigs may also be a risk factor.

The surveillance and control of HEV infection are very important around the world in order to decrease the knowledge gap in terms of its transmission and reservoirs, based on its zoonotic potential. In recent years, the One Health approach has high popularity and importance for specialists working on the topic of the HEV. Human and veterinary scientists (physicians) increasingly recognize the importance of working together on important zoonoses such as HEV infection. Professionals from all fields of science are responsible for controlling and dealing with this infection. Of course, it is recommended that the efforts of scientists be supported by adequate and timely policies and programs led by international and national health authorities and organizations.
